# Cisternostomy Facilitates Clearance of Metabolic Waste from Cerebrospinal Fluid in Patients with Severe Brain Injury

**DOI:** 10.14336/AD.2025.0738

**Published:** 2026-10-15

**Authors:** Weiwei He, Shuai Gao, Lulu Du, Tangrui Han, Di Yao, Hao Wu, Qiang Li, Feixiang Li, Woo-ping Ge, Yonghong Wang

**Affiliations:** ^1^Department of Basic Medical Science, Capital Medical University, Beijing 100069, China.; ^2^Chinese Institute for Brain Research, Beijing, Beijing 102206, China.; ^3^Beijing Institute for Brain Research, Chinese Academy of Medical Sciences & Peking Union Medical College, Beijing 102206, China.; ^4^Department of Neurosurgery, Shanxi Bethune Hospital, Shanxi Academy of Medical Sciences, Tongji Shanxi Hospital, Third Hospital of Shanxi Medical University, Taiyuan 030032, China.; ^5^Shanxi Coal Central Hospital, Taiyuan 030032, China.; ^6^Changping Laboratory, Beijing, 102206, China

**Keywords:** traumatic brain injury, cisternostomy, decompressive craniotomy, cerebrospinal fluid, metabolomics, metabolome, metabolite, hypertensive cerebral hemorrhage, subarachnoid hemorrhage

## Abstract

Decompressive craniotomy, a common intervention for traumatic brain injury (TBI), can fail to effectively alleviate patient symptoms. Cisternostomy, reported for cistern drainage in patients with severe brain injury (SBI), has shown efficacy in reducing intracranial pressure and clearing detritus resulting from brain hemorrhage. However, the mechanisms underlying their effectiveness remain largely unknown. Here, we utilized non-targeted metabolomics to analyze cerebrospinal fluid (CSF) from cisterns alongside peripheral blood samples from SBI patients undergoing cisternostomy. Through a systematic comparison of the cisternal cerebrospinal fluid and blood plasma metabolomes, we identified multiple blood-enriched metabolites, including betaine, triethanolamine, and proline, that were efficiently cleared during the early stage of SBI. Notably, two metabolites linked to arginine metabolism and the urea cycle, N8-acetylspermidine and N-acetylputrescine, showed significant reductions that correlated with improvements in the Glasgow Coma Scale (GCS). Our findings indicate that cisternostomy effectively removes blood-derived substances and aids the recovery of patients with early-stage brain injury.

## INTRODUCTION

The brain vasculature is uniquely characterized by the protective blood-brain barrier (BBB), which serves as the critical interface between the bloodstream and central nervous system (CNS). The BBB selectively filters metabolites between the blood and cerebrospinal fluid (CSF), and this mechanism is essential for maintaining CNS homeostasis [1]. CSF is predominantly produced by the choroid plexus, and it circulates throughout the CNS and is subsequently drained via arachnoid granulations and the glymphatic system [1, 2]. Substances that penetrate the BBB and reach brain tissue help regulate both the physical and chemical environment of the CNS; hence, any alteration or disruption of the BBB may expose the brain to harmful substances from the blood, which can lead to neuronal and glial dysfunction and ultimately cause brain damage. Thus, controlling the exchange of substances between the blood and the brain parenchyma is essential for brain function and protection [3-5].

Traumatic brain injury (TBI) is a major cause of disability and death worldwide, affecting an estimated 50 million people annually [6-8]. TBI can cause diffuse brain injury, which typically results from exposure of the head to a sudden accelerating or decelerating force. This can lead to a spectrum of pathological outcomes, including axonal injury, cerebral swelling, contusions, lacerations, and intracranial hemorrhage [9-11]. TBI is often accompanied by disruption of the BBB, thus impairing the transport of essential nutrients from the blood to the brain parenchyma [12]. Additionally, any compromise in BBB may facilitate the infiltration of blood-derived toxins, inflammatory molecules, and immune cells into the brain tissue [13-16]. This breach can trigger extensive neuroinflammation, oxidative stress, and ultimately neuronal damage [17, 18]. Consequently, BBB disruption or damage to the vasculature may lead to the development of brain edema, elevated intracranial pressure, or impaired cerebral autoregulation [14, 15, 19]. These pathological changes interfere with normal neuronal function and connectivity, ultimately resulting in diverse forms of brain dysfunction including cognitive deficits [15, 20], motor impairments [21, 22], and increased susceptibility to neurodegenerative diseases [23-27]. Therefore, reestablishing the integrity of the BBB as soon as possible after TBI would restore its ability to clear potentially noxious or exogenous substances from the brain, help prevent secondary brain injury, and promote recovery [1].

Decompressive craniectomy is a commonly performed neurosurgical procedure aimed at reducing intracranial pressure in patients with TBI [28] and other brain injuries. Although it relieves the pressure by creating physical space in which the brain can expand, this intervention does not accelerate the removal of blood-derived substances that infiltrate the CSF post-injury [29]. Perhaps for this reason, decompressive craniectomy has not consistently shown efficacy in treating cerebral edema, improving CSF circulation, or enhancing glymphatic system clearance [30]. Furthermore, due to the risks associated with bone flap loss, many patients require cranioplasty after the initial procedure [31]. Therefore, there is an urgent need to develop novel treatment modalities for TBI [29].

Since the cisternostomy procedure was reported [32-34], it is now used in the acute phase of severe brain injury (SBI) to mitigate intracranial pressure by creating a stoma in the intracranial basal cisterns, facilitating CSF drainage [35, 36]. This approach aims to prevent edema and thus secondary brain injury [19, 37]. However, the widespread adoption of cisternostomy has been limited, primarily due to uncertainties regarding the relationship between the efficacy of the procedure and the underlying mechanisms [36, 38, 39]. Additionally, the ability of the procedure to remove harmful substances (including metabolites) from the CSF—particularly blood-derived substances following cerebral hemorrhage—remains uncertain [40].

To address the challenges limiting the widespread use of cisternostomy for SBI and evaluate its efficacy, we performed a systematic metabolomic analysis of CSF and blood plasma samples (acquired 1-14 days post-injury) from patients who experienced severe brain injury. Our results demonstrate that cisternostomy, when administered during the acute stage after SBI, significantly reduced the levels of substances including metabolites from CSF and those enriched in blood. These findings provide evidence that cisternostomy provides substantial clinical benefit for managing SBI.

## MATERIALS AND METHODS

### Patient selection

Fifteen patients with severe craniocerebral injury presenting to the Department of Neurosurgery at Bethune Hospital in Shanxi who required surgical management and met the inclusion criteria were enrolled in the study (Protocol, YXLL-2018-05). Patients were enrolled regardless of primary etiology (traumatic or spontaneous) provided they met the inclusion criteria, allowing us to test the generalizability of cisternostomy-mediated clearance (Supplementary Table 1). All participants signed an informed consent form. The inclusion criteria were as follows: (1) age > 18 years and < 70 years; (2) acute brain injury requiring surgery (except for simple epidural hematoma); (3) intracranial pressure > 20 mmHg before craniotomy; (4) no significant changes in vital signs and no surgical contraindications. Exclusion criteria were as follows: (1) concomitant severe underlying systemic illnesses such as abnormal coagulation functions, liver failure, or renal dysfunction; (2) any incidence of severe trauma, penetrating head injury, or preexisting disease; (3) any injury to the thoracic/abdominal cavities. All patients were admitted to the neurosurgical intensive care unit for continuous monitoring of intracranial pressure for one week after surgery, assisted breathing with endotracheal intubation and a ventilator, sedation and analgesia, and symptomatic supportive treatment.

### Classification of cisternal CSF and sample collection information

To evaluate alterations in CSF composition at different stages of the acute phase in patients who underwent cisternostomy, our study classified cisternal CSF samples based on GCS score, timing of sample collection, and CSF clarity. Early-stage CSF (ES-CSF) is defined as samples collected on postoperative days 1-7 (which cover the acute stage usually people used clinically), characterized by bloody or xanthochromic appearance (consistent with red blood cell lysis) and corresponding to the clinical acute phase. These samples were obtained from patients with GCS scores 3-8, reflecting severe neurological impairment. Late-stage clear CSF (LS-CSF) refers to samples collected on days 8-14, which presented as crystal clear, indicating resolution of hemorrhage/ hemolysis. These samples correspond to the recovery phase, with patients achieving GCS scores 9-15, reflecting neurological improvement.

We collected a total of 370 samples from 15 patients (see Supplementary Table for detailed information), including 316 CSF samples and 54 blood samples. In some cases, both CSF and blood samples were collected from the same patient on the same day. Different subsets of samples were used for specific analyses, as detailed in the corresponding sections.

### Collection of peripheral plasma and cisternal CSF

CSF samples were collected from patients via basal cistern drainage every 12 h for a total of 14 times following the completion of surgery (catheter model: 18G ventricular catheter; insertion depth: 3-5 mm into basal cisterns; drainage rate: 5-8 mL/h). Clinical data, including intracranial pressure and GCS score were recorded. Strict anti-infection protocols implemented after surgery were as follows: (1) daily monitoring: body temperature, CSF appearance; (2) CSF testing: Cell count/biochemistry on post-operative days 3 and 7 for all patients; cultures in the case of suspected infection (fever >38.5°C + CSF WBC >100/μL); (3) antibiotics: A single dose of preoperative cefazolin (2g IV) was administered; (4) CSF leukocytes were monitored and consistently measured <5/μL (normal range) despite high RBC counts; (5) CSF glucose was monitored and found to be normal (mean 3.8 mmol/L), supporting the conclusion that no bacterial metabolism was occurring. (6) all 32 CSF cultures (sent when turbidity and/or fever was present) were negative.

Blood samples were collected from peripheral veins. Both CSF and blood were stored in sterile, heparin-coated anti-coagulation tubes. The CSF and plasma supernatants were separated by centrifugation at 1,200 × *g* for 15 min at 4°C. All aliquots were stored at -80°C until analysis.

### Metabolite extraction for metabolomic analysis

CSF and plasma samples were thawed at 4 °C. Subsequently, 30 μL CSF and 15 μL plasma were transferred into Eppendorf tubes containing 240 μL of ice-cold 90% acetonitrile (ACN)/water (v/v, HPLC-grade, Merck, catalog no. 1.00030.4008) and 185 μL of ice-cold 86.5% ACN/water (v/v), respectively. Metabolites were extracted, resulting in a final ACN concentration of 80%. After centrifugation at 15,000 × *g* for 15 min at 4°C, 200 μL CSF supernatant and 160 μL plasma supernatant (corresponding to metabolites extracted from 30 μL CSF and 15 μL plasma, respectively) were dried using a centrifugal concentrator (SpeedVac) at room temperature to obtain a pellet. The pellet was subsequently stored at -80°C for metabolomic profiling analysis. Additionally, two types of quality-control pools were prepared by combining equal volumes of CSF and plasma samples, which were then evenly spaced throughout the injection sequence. The samples were re-dissolved in 160 μL of 80% ACN and transferred to a Q Exactive (Thermo Fisher Scientific, San Jose, CA) Orbitrap mass spectrometer (MS) for non-targeted (untargetd) analysis.

### Non-targeted metabolomic measurement with LC-MS

The metabolites were separated chromatographically using an Agilent Infinity Lab Poroshell 120 HILIC-Z column (2.1 × 100 mm, 2.7 μm) equipped with a PEEK liner and detected using a Q Exactive mass spectrometer. The mobile phases for positive ionization mode were as follows: phase A, water containing 10 mM ammonium formate (Sigma, catalog no. 70221), and phase B, HPLC-grade ACN containing 10 mM ammonium formate; for negative ionization mode, mobile phases were: phase A, water containing 10 mM ammonium acetate (Sigma, catalog no. 73594), and phase B, ACN containing 10 mM ammonium acetate. The solvent gradient was as follows: 0-4 min, 100% B to 84% B; 4-11 min, 84% B to 40% B; 11-12 min, 40% B; 12-13 min, 40% B to 100% B; and 13-17 min, 100% B. The injection volume was 3 μL, with a flow rate of 0.4 mL/min, and the column temperature was 35 °C. All samples were stored at 4°C throughout the analysis period. The temperature of the injector was set at 4 °C, which was consistent with the sample storage temperature, aiming to avoid the degradation of metabolites.

Both positive and negative ion scanning modes were employed with the ultraperformance liquid chromate-graphy (UPLC) system to acquire high-quality spectra for the samples, which were interfaced with a Q Exactive Orbitrap mass spectrometer equipped with an electrospray ionization source. For MS1 detection, the automatic gain control (AGC) target was set to 3,000,000 with a maximum injection time of 100 ms. The resolution was set to 70,000 in full-scan MS mode, with a scan mass range of 60-900 m/z. MS2 detection was performed at a resolution of 17,500, covering a mass range of 60-400 m/z and 350-900 m/z for the mixed quality-control samples. The AGC target was set to 50,000 with a maximum injection time of 80 ms, and the minimum AGC target was set to 1,000, with a dynamic exclusion duration of 6 s. Mass spectrometry ion source parameters: spray voltage 3.5 kV (positive mode) and 3.2 kV (negative mode); Fragmentation was performed using stepped collision energies of 20, 30 and 40 Normalized Collision Energy (NCE) units.

### Data preprocessing and statistical analysis

The collected LC-MS/MS raw data files were imported into Compound Discoverer 3.2 software (Thermo Fisher Scientific) to obtain matched peak data. A total of 28,244 and 14,637 features were detected in positive and negative ionization modes, respectively. To ensure data quality, data cleaning was performed as follows: First, an initial precursor mass deviation of up to 5 ppm and a fragment mass deviation of up to 20 ppm were allowed. Next, the signal-to-noise ratio was calculated as the ratio of the average intensity of all samples to that of the blank, and it was maintained above 2. Additionally, the average intensity of all samples was required to be above 3 × 10^4^. After applying these screening criteria, 13,114 features were retained.

The peak intensity data, representing the abundance of metabolites in each sample, were normalized using Z-score transformation. The selected compounds were identified using the Human Metabolome Database (HMDB) database and ion fragments obtained from the mass spectrometry analysis. Differential metabolite data were analyzed using one-way analysis of variance with GraphPad Prism 9.0 software. Pathway enrichment analysis was performed through the MetaboAnalyst platform, and related pathways for the differential metabolites were analyzed using the KEGG online database.

## RESULTS

### Characterizing the difference in metabolic profiles between blood and clear cisternal CSF

To obtain and compare the metabolomes of blood and clear cisternal CSF from early-stage patients, peripheral blood plasma (n = 20 samples from 10 subjects) and LS-CSF (n = 20 samples from 5 subjects; clear cisternal was defined as cisternal CSF from patients with high GCS scores from 9-15 and without hemolysis) were collected for non-targeted metabolomic profiling (Fig. 1A). Overall, there were substantial differences between the metabolomes of blood and LS-CSF (Fig. 1B). A total of 2,159 metabolic features (briefly, we used metabolites in subsequent text) were observed to be enriched in blood, whereas 4,164 were enriched in LS-CSF (Fig. 1C, filtered by fold change, FC >1.5 and an adjusted *p* < 0.05); these accounted for 16.46% and 31.75%, respectively, of the 13,114 metabolites we measured (Fig. 1D). Subsequent analysis of the chemical species among these enriched metabolites revealed a prevalence of lipids, particularly various glycerophospholipids, in blood. In contrast, water-soluble metabolites were relatively enriched in clear cisternal CSF (Fig. 1E). We next conducted a biochemical pathway analysis of the enriched metabolites and found that the linoleic acid pathway was specifically enriched in blood (Supplementary Fig. 1A). Additionally, pathways associated with amino acids, coenzymes, and vitamins (e.g., nicotinate and nicotinamide metabolism pathway) were found to be enriched in cisternal CSF (Supplementary Fig. 1B). We further performed a KEGG pathway enrichment analysis of the metabolites enriched in blood and CSF (Supplementary Fig. 2-4). A substantial number of metabolic pathways were significantly enriched in blood, including lipid metabolism-related pathways such as choline and glycerophospholipids. In contrast, certain other subclasses of metabolites were enriched in cisternal CSF, including pathways related to the biosynthesis of tyrosine, arginine and proline and the histidine metabolism pathway (Supplementary Fig. 2).

### Characterizing the differences in molecular species between LS-CSF and cisternal CSF in samples from early-stage patients

To elucidate the molecular mechanisms by which cisternostomy contributes to the recovery of patients with SBI, we conducted a comparative metabolomic analysis of CSF collected at two different stages of recovery. Specifically, we examined the differences between cisternal CSF obtained during the early stage of brain injury (ES-CSF, n=16 samples from 7 subjects) and LS-CSF collected during the late stage (n=20 samples from 5 subjects) from patients who underwent cisternostomy. Significant differences were observed between the metabolomes of ES-CSF and LS-CSF (Fig. 2A, FC > 1.5, adjusted *p* < 0.05). Of the 13,114 metabolites analyzed, 10.49% (1,376 metabolites) were enriched in ES-CSF, whereas 13.07% (1,714 metabolites) were enriched in LS-CSF (Fig. 2B).

To investigate the proportion of blood-derived metabolites present in ES-CSF, we integrated the blood-enriched metabolites identified earlier with the metabolites enriched in ES-CSF (Fig. 1). Two-thirds of the metabolites (n = 918) enriched in ES-CSF overlapped with the blood-enriched metabolites (Fig. 2C and D), suggesting that a substantial portion of the metabolites in ES-CSF may have originated from blood clots or were a consequence of cerebral hemorrhage. Among the blood-derived metabolites, lipophilic substances were more prevalent in ES-CSF, whereas hydrophilic substances were relatively enriched in LS-CSF (Supplementary Fig. 5 and Fig. 2E). The above observations revealed similar enrichment characteristics to the above differential metabolites between blood and LS-CSF (Fig. 1E).

**Figure 1.**
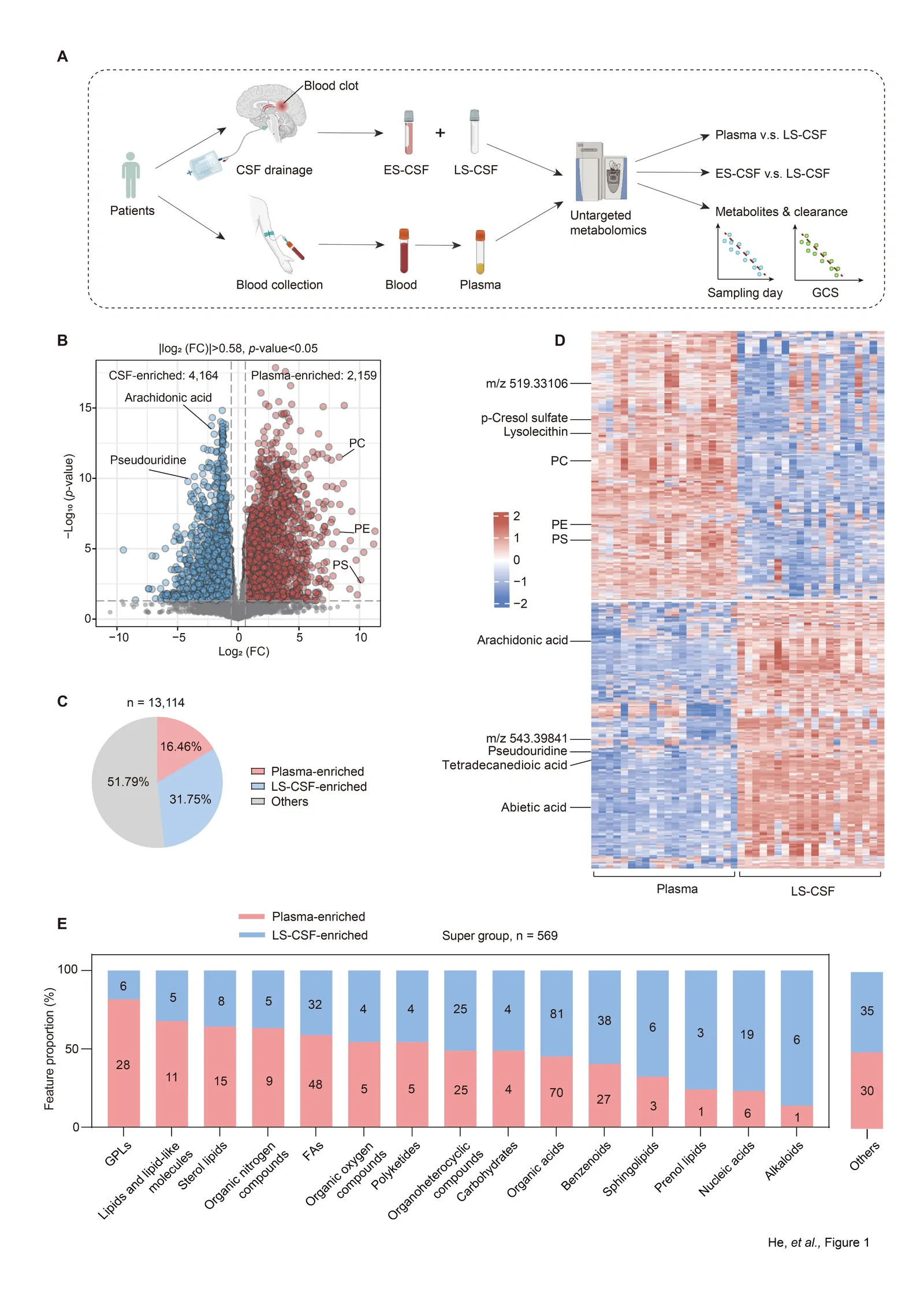
**Comparison of metabolomes of peripheral plasma and clear cisternal cerebrospinal fluid (CSF) from patients with SBI**. (**A**) Schematic illustration demonstrating the strategy for collecting plasma and CSF samples, followed by metabolomic measurements and analysis. (**B**) Volcano plot showing the most significantly different metabolic features between plasma and cisternal CSF (p < 0.05, |log_2_FC| > 0.58, i.e., FC > 1.5 or FC < 0.67). Plasma samples: n=20 (from 10 patients); LS-CSF samples: n=20 (from 5 patients). Statistical tests: two-tailed Student’s t-test with Benjamini-Hochberg FDR correction (adjusted for 13,114 metabolic features). *FC*: fold change. (**C**) Percentage of differential metabolites between plasma and CSF samples among all metabolites measured. (**D**) Heatmap of the top 3,000 differential metabolic features between plasma and LS-CSF. Data were obtained using non-targeted metabolomic analysis with LC-MS (see *Methods* for details). The color scale represents the levels of standardized metabolites. (**E**) Enrichment analysis of metabolites from different sub-clusters. The metabolite clusters were categorized based on chemical structures from differential and annotated blood-enriched and cisternal CSF-enriched metabolites. A total of 569 annotated metabolites were identified among all differential metabolites. PC: phosphatidylcholine; PE: phosphatidylethanolamine; PS: phosphatidylserine; GPL: glycerophospholipid; FA, fatty acid.

To determine the likelihood that blood-enriched metabolites were discharged into ES-CSF, the 2,159 blood-enriched metabolites (Fig. 1C) were divided into 20 equivalent subgroups, i.e., each group contained 108 (~5%) metabolites. These subgroups were plotted in order of descending relative abundance. Simultaneously, the 918 metabolites that were enriched in both blood and ES-CSF were also ranked in descending order based on abundance in ES-CSF and then divided into 20 subgroups of 46 metabolites (5%) each. We then compared the distribution of these 46 ES-CSF-enriched metabolites with the 108 blood metabolites in the corresponding ordinal section. Intriguingly, this analysis revealed a correlation between decreased abundance of a metabolite in ES-CSF and its decreased metabolite in blood (Fig. 2F). Moreover, 57% of potential blood-derived metabolites in ES-CSF were concentrated in the first 6 sections, representing the top 30% of blood-enriched metabolites (Fig. 2F). These results indicated that these metabolites, which were enriched in ES-CSF compared with LS-CSF, were likely derived from blood clots or leaked blood. The results also indicated that cisternal CSF drainage during cisternostomy led to the gradual discharge of abundant blood-derived substances.

To further assess the overlapping metabolites among LS-CSF, ES-CSF, and blood, we compared the abundance of 88 blood-enriched metabolites and examined their abundance in ES-CSF. These blood-enriched metabolites could be categorized into three distinct subgroups (Supplementary Fig. 6). The first group comprised 49 metabolites that were most abundant in blood and least abundant in LS-CSF. This group accounted for 55.68% of the 88 total blood-enriched metabolites, including carnitine, orinithine, L-alanine. Within this group, certain metabolites showed a smaller disparity in abundance between ES-CSF and LS-CSF. The second group comprised 21 members (23.86% of the total 88) including lysine, proline and m/z 202.04508 (Fig. 2G and H) and was characterized by having the highest abundance in blood but also having high abundance in ES-CSF. The third group comprised 18 members characterized by having the highest abundance in ES-CSF including triethanolamine betaine and choline, constituting 20.45% of the total (Fig. 2I, J and K), indicating that certain blood-derived metabolites may have become more concentrated upon entry into CSF due to its limited volume. The second and third groups of metabolites likely originated from blood and entered the CSF as a result of brain injury-induced hemorrhage. The second group, in particular, likely originated from blood but became more concentrated upon entering the cisternal CSF due to the limited ventricular space.

### Identifying metabolites cleared from the brain during cisternostomy

To further investigate the efficacy of cisternostomy for clearing blood-derived metabolites from ES-CSF following brain injury, we conducted a correlation analysis between GCS score, the timing of CSF sample collection from cistern drainage, and the abundance of 13,114 metabolites obtained from non-targeted metabolomic measurements (Fig. 3A and B). A significant number of metabolites correlated positively with sampling time and/or GCS score, representing 19.03% (2,496 metabolites) and 5.72% (598 metabolites), respectively, of the total 13,114 metabolites. Conversely, some metabolites correlated negatively with sampling time and/or GCS score, accounting for 4.56% (750 metabolites) and 2.13% (279 metabolites), respectively, of the total (Fig. 3A and B).

To further elucidate the characteristics of the group of metabolites for which abundance correlated with cisternal CSF drainage (i.e., sampling time progression) and patients’ recovery (i.e., GCS score improvement), we analyzed their chemical properties. Specifically, we examined the distribution of RT (Fig. 3C and D) and molecular weight (Fig. 3E and F). The chemical properties of substances that correlated positively with sampling time and GCS (Fig. 3A and B) differed significantly from those that correlated negatively, particularly in RT distribution (Fig. 3C and D). RTs of positively correlated substances (Sam-pos and GCS-pos) were generally short (0-3 min). Since HILIC columns retain polar molecules longer, this suggested positively correlated substances are more lipophilic. In contrast, negatively correlated substances showed a uniform RT distribution (0-6 min), indicating higher polarity.

**Figure 2.**
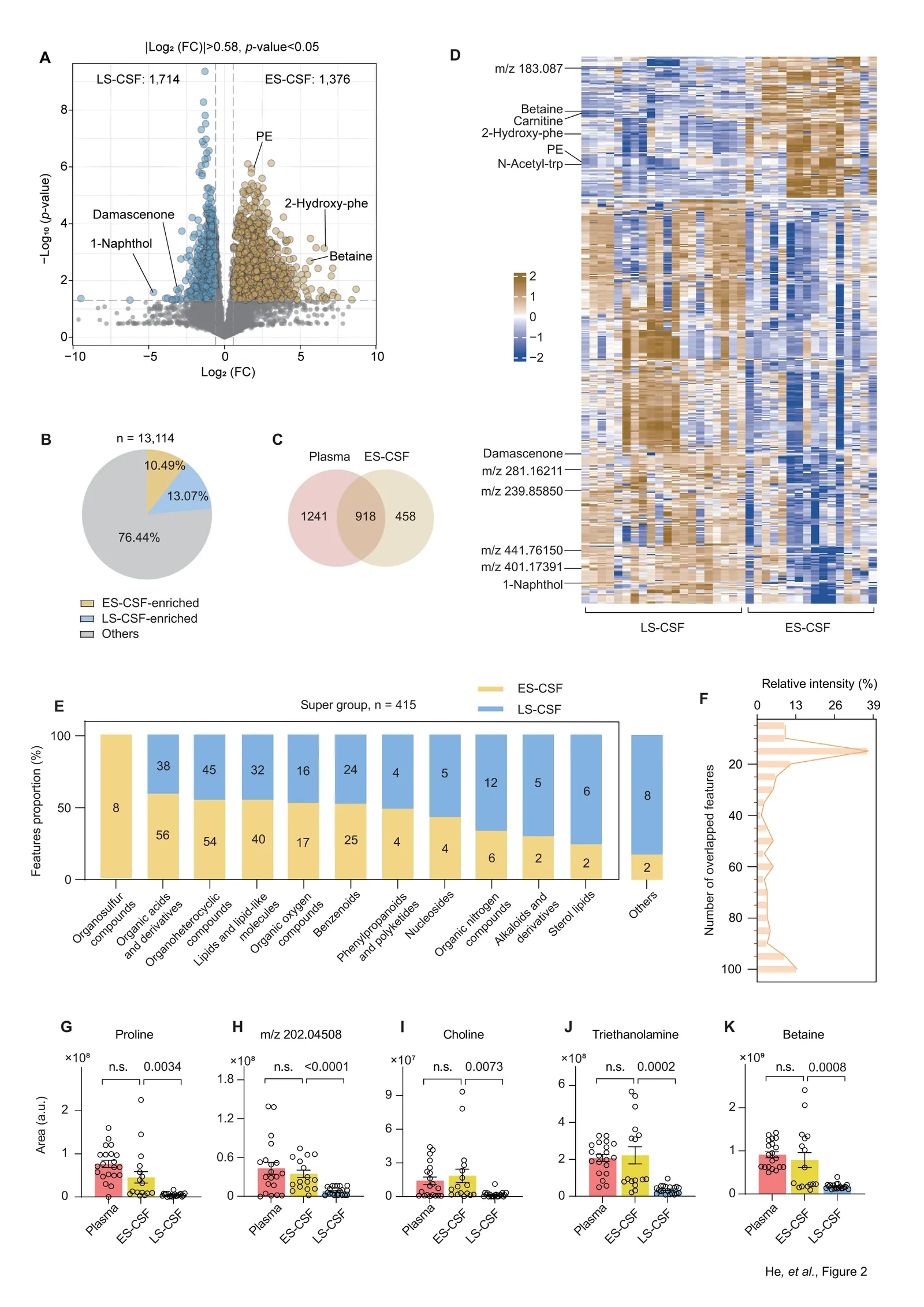
**Metabolomic comparison of early-stage cisternal CSF (ES-CSF) and clear cisternal CSF**. (**D**) Heatmap of the top 3,000 differential metabolic features between ES-CSF and LS-CSF. The color scale represents the levels of standardized metabolites. (**A**) Volcano plot showing the most significantly different features and corresponding metabolites between ES-CSF and LS-CSF (p < 0.05, |log_2_FC| > 0.58, i.e., FC > 1.5 or FC < 0.67). (**B**) Percentage of metabolites for which differences were statistically significant between ES-CSF and LS-CSF. (**C**) Venn diagram illustrating the overlap between blood-enriched and ES-CSF-enriched metabolic features. Blood-enriched features were identified from plasma (n=20 samples from 10 patients) vs. LS-CSF (n=20 samples from 5 patients); ES-CSF-enriched features were identified from ES-CSF (n=16 samples from 7 patients) vs. LS-CSF (n=20 samples from 5 patients). A total of 918 overlapping features were detected. (**E**) Enrichment analysis of metabolites based on the chemical structures of metabolites that were enriched in ES-CSF or LS-CSF. A total of 415 annotated metabolites were divided into 12 distinct groups. (**F**) Proportion of ES-CSF-enriched features within blood-enriched features, categorized based on the ranking of relative intensity. (**G**) Area of five representative metabolites (betaine, triethanolamine, proline, m/z 202.04508, and choline) in peripheral plasma (n=20 samples from 10 patients), early-stage cisternal CSF (ES-CSF, n=16 samples from 7 patients), and late-stage clear cisternal CSF (LS-CSF, n=20 samples from 5 patients). Data are presented as dot plots to show individual values. Statistical significance was determined using two-tailed Student’s t-test, with Benjamini-Hochberg FDR correction for 5 metabolites (adjusted *p* < 0.05). No significant difference was observed between the blood and ES-CSF groups. After correction, the *p*-values between ES-CSF and LS-CSF were 0.0008, 0.0002, 0.0034, < 0.0001, and 0.0073, respectively. p-values < 0.01 were marked as ** and those < 0.001 as ***. PE: phosphatidylethanolamine; Trp: tryptophan.

Molecular weight (MW) distributions differed slightly between sampling time and GCS correlations (Fig. 3E and F). Substances positively correlated with sampling time were predominantly 600-800 Da, whereas negatively correlated ones were mainly <600 Da—suggesting larger MWs for positively correlated substances and smaller MWs for negatively correlated ones. For GCS correlations, most substances (both positive and negative) fell within 100-400 Da, with no significant MW differences.

We further observed that the abundances of specific metabolites consistently correlated with the increase of GCS score and sampling time. For instance, the abundances of m/z 304.21121, m/z 201.05650, N8-acetylspermidine and N-acetylputrescine correlated negatively with GCS score and sampling time (Fig. 3G-N). This suggested that the concentrations of these metabolites progressively decreased in conjunction with cisternal CSF drainage and recovery from brain injury. Conversely, the abundance of m/z 363.20636 correlated positively with both GCS score and sampling time, suggesting that its concentration progressively increased during drainage and recovery (Fig. 3O and S). We also found that the abundances of certain metabolites, including m/z 153.06578, butyryl-l-homoserine lactone and phosphatidylserine, were solely positively correlated with sampling time (Fig. 3P-R). In contrast, the abundances of m/z 244.22691, m/z 241.10609 and l-tartaric acid were exclusively positively correlated with GCS score (Fig. 3T-V).

### Clearance of metabolites by cisternostomy and its contribution to recovery

To determine whether cisternal CSF drainage during cisternostomy helps eliminate metabolic waste from the CSF of brain injury patients, we analyzed the correlation between the abundance of specific metabolites in cisternal CSF and GCS score. First, we identified a pool of metabolites that correlated negatively with GCS score (i.e., their levels decreased gradually with increasing GCS score) and with sampling time (i.e., characterized by a gradual decrease in abundance over time). The metabolites that correlated negatively with GCS score significantly overlapped with those enriched in blood (top 1,000 blood-enriched metabolites; Fig. 4A and B) and clear cisternal CSF (Fig. 4C and D). This analysis revealed that, among the 598 substances that correlated negatively with sampling time, 278 overlapped with blood-enriched metabolites (46.5%, Fig. 4A), whereas only 47 overlapped with CSF-enriched substances (4.5%, Fig. 4C). Similarly, among the 279 metabolites that correlated negatively with GCS score, 67 (46.7%) overlapped with blood-enriched substances (11.2%, Fig. 4B), whereas a larger number overlapped with CSF-enriched substances (Fig. 4D). These findings indicated that some of these negatively correlated metabolites may have originated from blood. These metabolites were gradually excreted via CSF drainage during cisternostomy, leading to a decrease in blood-enriched metabolites in the CSF and contributing to recovery from brain injury.

Furthermore, we separately compared the number of features within these overlapping datasets against their relative abundance in blood and CSF. The abundance of enriched substances in blood and cisternal CSF decreased with increasing GCS score and sampling time, and the abundance of substances in the overlaps had an overall downward trend. This suggested that the abundance of certain blood-derived metabolites in the CSF diminished progressively and may have been cleared through the drainage process.

Additionally, we compared the 598 metabolites that correlated negatively with CSF sampling time with the 279 metabolites that correlated negatively with GCS score. We found that 32 metabolites were common to both sets, indicating that the abundance of these substances decreased with both sampling time and the improvement in GCS score. Further analysis of these 32 metabolites revealed that 23 (71.9%) were among the top 1,000 highly enriched compounds in blood. Notably, these metabolites predominantly were within the top 50% in terms of abundance (Fig. 4E). These findings suggested that certain negatively correlated substances may have originated from blood clots in ES-CSF following brain injury. Moreover, these substances appeared to be progressively eliminated through cisternostomy, facilitated by CSF drainage and the patient's subsequent recovery.

**Figure 3.**
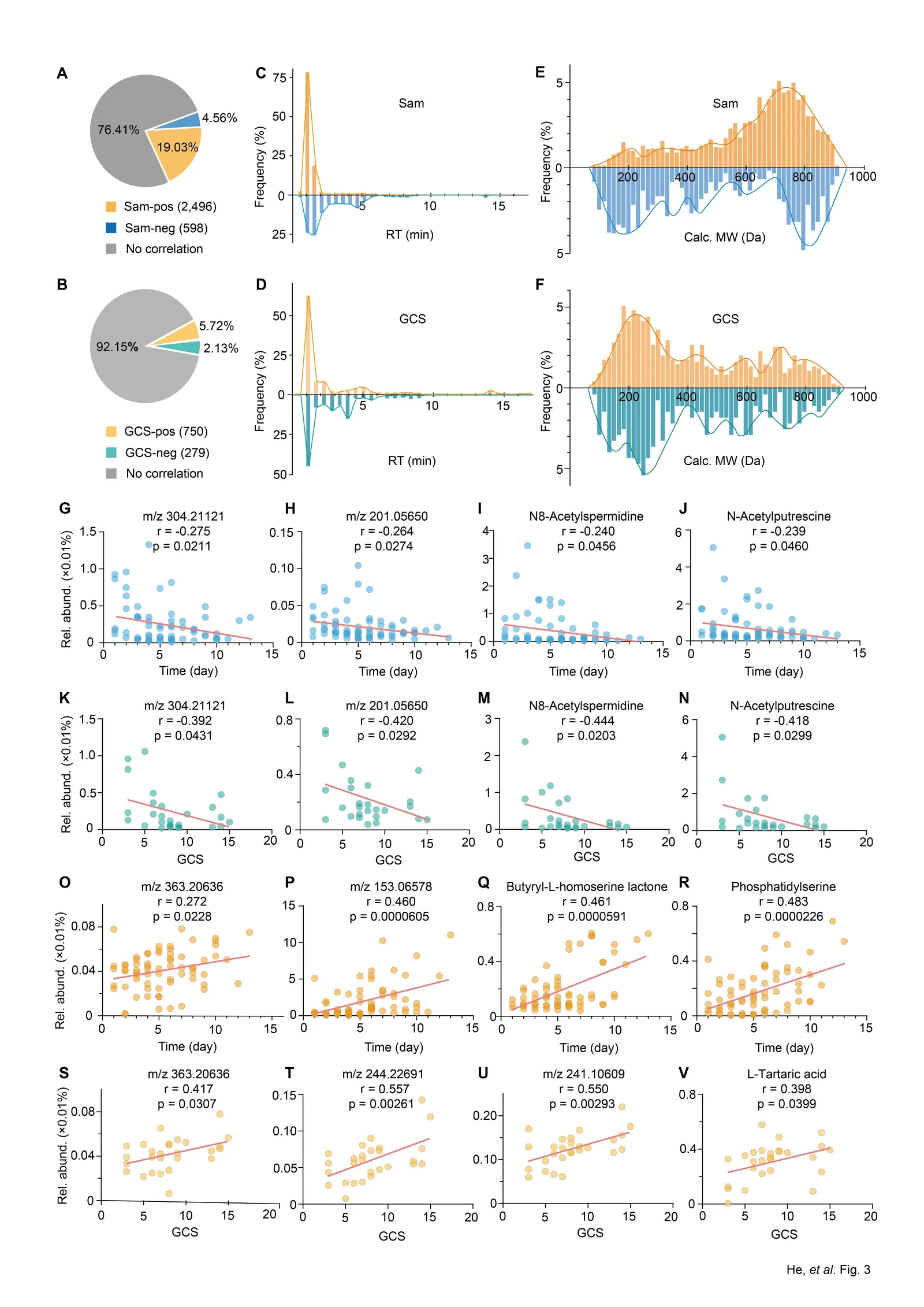
**Identification of metabolites correlated with functional brain recovery in TBI patients**. (**A**) Correlation analysis between cisternal metabolites and sampling day. Positively correlated metabolites (Sam-up, *r* > 0.35, *p* < 0.05) are shown in orange, accounting for 19.03% of the total (2,496 features). Negatively correlated metabolites (Sam-down, *r* < -0.35, *p* < 0.05) are shown in blue, accounting for 4.56% of the total (598 features). The remaining 76.41% of features exhibited no significant correlation. (**B**) Correlation analysis between cisternal metabolites and GCS score. Positively correlated metabolites (GCS-up, *r* > 0.35, *p* < 0.05) are shown in yellow, accounting for 5.72% of the total (750 features). Negatively correlated metabolites (GCS-down, *r* < -0.35, *p* < 0.05) are shown in green, accounting for 2.13% of the total (279 features). The remaining 92.15% of features exhibited no significant correlation. (C, D) Distribution plots of retention time (RT, liquid chromatography) of metabolites correlated with sampling day (Sam-up and Sam-down) and GCS score (GCS-up and GCS-down). (E, F) Distribution plots of the relative molecular weights of metabolites correlated with sampling day and GCS score. (**G-J**) Representative metabolites that correlated negatively with sampling day, including *m/z* 304.21121, *m/z* 201.05650, N8-acetylspermidine, and N-acetylputrescine. The *p*-value was calculated using Pearson correlation analysis, with Benjamini-Hochberg correction for 13,114 metabolic features. (**K-N**) Representative metabolites that correlated negatively with GCS score, including *m/z* 304.21121, *m/z* 201.05650, N8-acetylspermidine, and N-acetylputrescine. The *p*-value was calculated using Pearson correlation analysis, with Benjamini-Hochberg correction for 13,114 metabolic features. G-J, O-R, n = 70 CSF samples collected from 15 SBI patients over days 1-13. Each patient contributed 2-8 samples, with one sample collected per day per patient. K-N, S-V, n = 27 CSF samples collected from 15 patients with GCS scores ranging from 3 to 15. Each patient contributed 1-4 samples, with one sample corresponding to each GCS score per patient. (**O-R**) Representative metabolites that correlated positively with sampling day, including *m/z* 363.20636, *m/z* 153.06578, butyryl-l-homoserine lactone, and phosphatidylserine. The *p*-value was calculated using Pearson correlation analysis, with Benjamini-Hochberg correction for 13,114 metabolic features. (**S-V**) Representative metabolites that correlated positively with GCS score, including *m/z* 363.20636, *m/z* 153.06578, butyryl-l-homoserine lactone, and phosphatidylserine. The *p*-value was calculated using Pearson correlation analysis, with Benjamini-Hochberg correction for 13,114 metabolic features.

### Conclusion

In this study, we collected a total of 316 cisternal CSF and 54 peripheral venous blood samples from 15 patients undergoing cisternostomy-based CSF drainage treatment for SBI. This non-targeted metabolomic study represents the first comprehensive comparison and correlation analysis of the metabolic characteristics of blood and clear cisternal CSF. Our results reveal that blood in the samples was predominantly enriched with lipophilic compounds of higher molecular weight, including phosphatidyl-choline, phosphatidylethanolamine, and other glycerophospholipids, whereas cisternal CSF contained a higher proportion of hydrophilic metabolites of smaller molecular weight. Additionally, a significant proportion of metabolites present in CSF during the acute injury phase displayed distinct characteristics compared to those observed in LS-CSF during the recovery phase. Notably, many metabolites enriched in ES-CSF also were enriched in blood. This suggests that ES-CSF contains an abundance of blood-derived metabolites. Their elevated concentrations in the bloodstream likely increase the probability of infiltration into the brain parenchyma via blood clots, resulting in the formation of ES-CSF. Furthermore, some blood-derived metabolites may further accumulate upon entering the CSF.

Using GCS score and the duration of cisternal CSF drainage as key indicators of recovery of brain injury patients, we systematically analyzed the alterations in metabolite levels in cisternal CSF during cisternostomy. Metabolites that correlated positively with recovery were predominantly derived from CSF, whereas the negatively correlated metabolites, which decreased in abundance during drainage, tended to be blood-derived. Among the negatively correlated metabolites, 23 blood-enriched compounds gradually decreased in abundance in cisternal CSF as GCS score and sampling time increased. These metabolites, including N8-acetylspermidine and N-acetylputrescine, represent blood-derived compounds consistently excreted during recovery of patients with SBI. N8-Acetylspermidine overabundance has been implicated in myocardial cell death, ischemic cardiomyocyte injury, and subsequent cardiac dysfunction [41]. Both N8-acetylspermidine and N-acetylputrescine are classified as polyamines and have been detected in blood, saliva, urine, and CSF [42,43]. Polyamines are linked to arginine metabolism and the urea cycle. N-Acetylputrescine, which is primarily derived from amino-acid catabolism, is somewhat cytotoxic and is produced during cadaver decomposition [44]. It has also been suggested as a potential biomarker for Bachmann-Bupp syndrome (commonly known as BABS), a condition characterized by global developmental delays, hypotonia, nonspecific physical abnormalities, and behavioral issues, including autism spectrum disorder and attention-deficit hyperactivity disorder. Previous studies have shown that polyamines contribute to cardiac health as well as brain development and function but also promote aging [45-47]. Therefore, maintaining polyamine levels within a physiological range is critical, as dysregulation can exacerbate cancer, neurodegenerative diseases, and aging [48-52]. Elevated polyamine levels are particularly problematic in neurological disorders such as Parkinson’s disease and epilepsy, in which significantly higher concentrations have been detected in CSF [50, 51].

**Figure 4.**
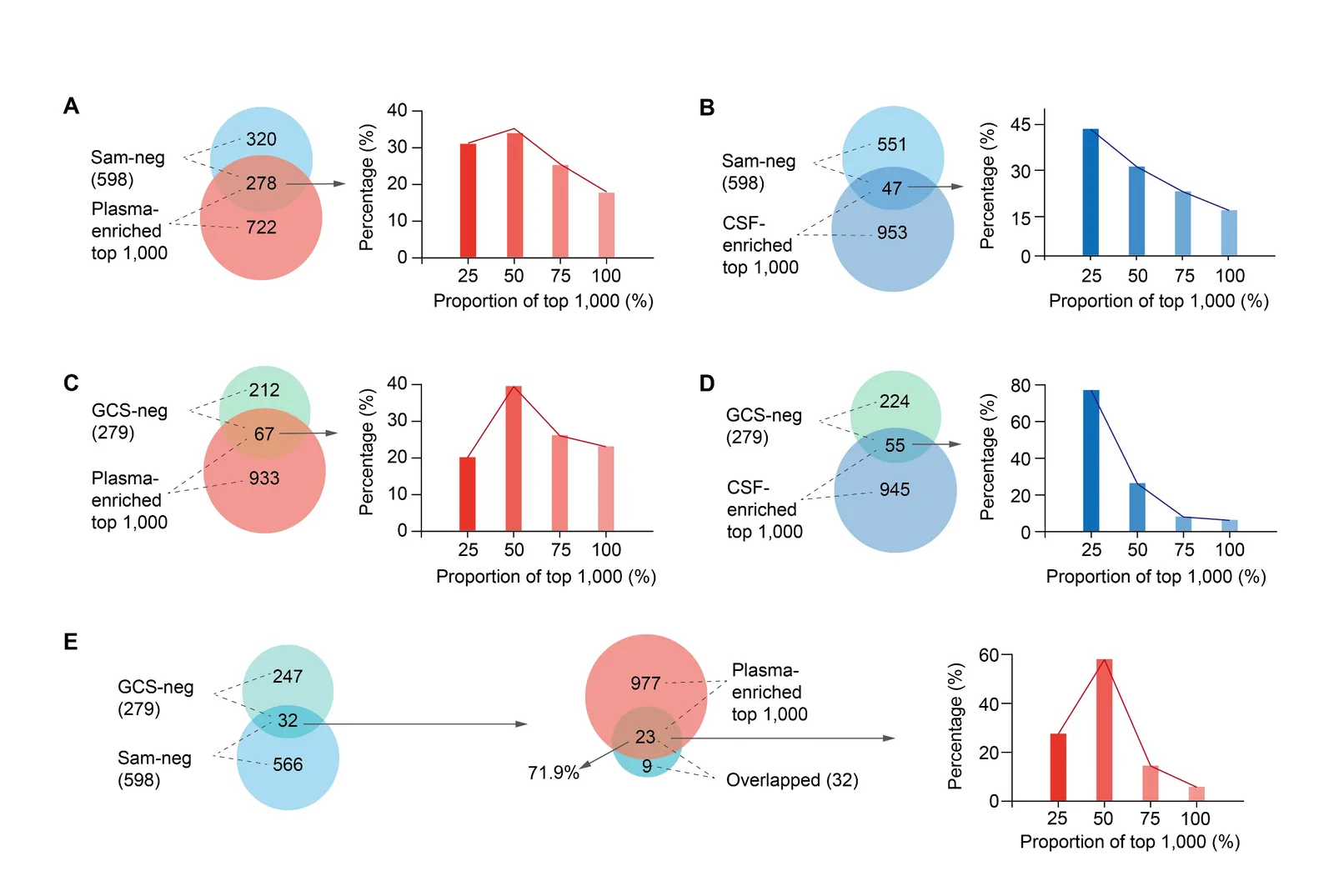
**Changes in metabolites in cisternal CSF assessed with analysis after cisternostomy**. (**A**) Overlap of 278 metabolites between the Sam-neg cluster (metabolites that correlated negatively with sampling day) and the top 1,000 plasma-enriched metabolites. The distribution of these shared features across the 25%, 50%, 75%, and 100% quartiles of the top 1,000 plasma-enriched metabolites was 28.8%, 32.7%, 23%, and 15.5%, respectively. (**B**) Overlap of 47 metabolites between the Sam-neg cluster and the top 1,000 CSF-enriched metabolites. The distribution of these shared features across the 25%, 50%, 75%, and 100% quartiles of the top 1,000 CSF-enriched metabolites was 40.4%, 27.7%, 19.1%, and 12.8%, respectively. (**C**) Overlap of 67 metabolites between the GCS-neg cluster (metabolites that correlated negatively with GCS score) and the top 1,000 plasma-enriched metabolites. The distribution of these shared features across the 25%, 50%, 75%, and 100% quartiles of the top 1,000 plasma-enriched metabolites was 17.9%, 37.3%, 23.9%, and 20.9%, respectively. (**D**) Overlap of 55 metabolites between the GCS-neg cluster and the top 1,000 CSF-enriched metabolites. The distribution of these shared features across the 25%, 50%, 75%, and 100% quartiles of the top 1,000 CSF-enriched metabolites was 72.7%, 21.9%, 3.6%, and 1.8%, respectively. (**E**) Overlap of 32 metabolites between the Sam-neg and GCS-neg clusters. Of these, 71.9% (23 metabolites) originated from the top 1,000 plasma-enriched metabolites. The distribution of these shared metabolites across the 25%, 50%, 75%, and 100% quartiles of the top 1,000 plasma-enriched metabolites was 26.1%, 56.5%, 13.1%, and 4.3%, respectively.

The clearance of polyamines (particularly N8-acetylspermidine and N-acetylputrescine) observed in this study likely has functional effects mediated through multiple mechanisms. First, excessive polyamine accumulation post-brain injury may induce neurotoxicity via reactive oxygen species (ROS) production, mitochondrial dysfunction, and neuronal apoptosis [48, 49]. These processes may be alleviated by the cisternostomy-mediated clearance of polyamines, consistent with the observed correlation between reduced polyamine levels and improved GCS scores. Second, polyamines are known to activate microglia and astrocytes, upregulating pro-inflammatory factors (e.g., TNF-α, IL-1β) that drive secondary brain injury; their removal may thus dampen neuroinflammatory cascades. As key intermediates in arginine metabolism and the urea cycle [42, 43], polyamine clearance could further help restore cerebral nitrogen balance, mitigating metabolic acidosis and supporting neuronal recovery. Collectively, these mechanisms suggest that cisternostomy-induced polyamine clearance contributes to neuroprotection by alleviating toxicity, regulating inflammation, and normalizing metabolic pathways.

Notably, the temporal changes in metabolite levels observed in this study are unlikely to merely reflect the natural recovery of metabolism. First, the rapid clearance of blood-derived metabolites (such as betaine, triethanolamine, and proline) is highly synchronized with the duration of cerebrospinal fluid (CSF) drainage from the cisterns. This change directly corresponds to the initiation of cisternostomy, rather than the gradually worsening metabolic patterns typically seen during the acute phase of SBI. Second, the decline of certain key metabolites (such as N8-acetylspermidine and N-acetylputrescine) is not only clearly time-dependent but also significantly correlated with improvements in the GCS score. This suggests that these metabolic changes are associated with neurological recovery, rather than being random fluctuations over time. Finally, metabolites with relatively long natural clearance half-lives, such as N8-acetylspermidine, exhibit a rapid decline during post-surgical CSF drainage, further supporting that their clearance is primarily driven by the cisternostomy procedure itself rather than natural recovery.

GCS scores are primarily used for neurological function assessment during the early stage, including preoperative, intraoperative, and early postoperative periods (usually within one month). In contrast, Scale Extension (GOSE) scores and cognitive assessments are more commonly applied for long-term prognostic evaluation, typically 2-3 months after surgery. Considering that the sampling time points in our study were mainly within one month after surgery, we therefore prioritized the use of GCS scores. In future studies, we plan to integrate both GOSE scores and cognitive assessments to obtain a more comprehensive understanding of the relationship between metabolic alterations and clinical outcomes.

Since decompressive craniectomy does not involve opening the skull base or the cisternal region, it is technically difficult—if not impossible—to obtain CSF from the cistern for comparison. Although some patients may undergo lumbar puncture for CSF collection, it is generally not feasible to perform multiple serial samplings within a short time frame. In contrast, ventricular fenestration allows continuous CSF drainage through catheters placed in the ventricles or cisterns, providing excellent conditions for repeated short-interval sampling. Therefore, this surgical procedure offers a unique opportunity to investigate CSF metabolic changes during both the early and late stages (in the present work, n=370 samples from 15 patients). We plan to explore in future studies how to compare CSF metabolism between these two surgical procedures.

Our findings suggest that cisternostomy combined with CSF drainage can effectively clear blood-derived metabolites, contributing to the removal of metabolic waste. However, this study has limitations due to the complex nature of the surgical procedure involved and the challenge of collecting uniform CSF samples from patient cisterns. The inclusion of only a small cohort of patients with SBI—despite yielding 316 CSF and 54 blood samples—may have introduced potential biases due to the sample size. Furthermore, the cohort included patients with varying primary etiologies, such as hypertensive cerebral hemorrhage (HICH), head trauma (CT), subarachnoid hemorrhage due to aneurysm (SAH). This heterogeneity, coupled with the aforementioned challenge associated with CSF collection, necessitates further studies to compare the efficacy of cisternostomy treatment across diverse etiologies. Although varying etiologies are present in our cohort, sensitivity analysis supported consistent metabolite clearance patterns across major subgroups (traumatic vs. hypertensive), suggesting the clearance mechanism is primarily dependent on blood-CSF barrier disruption rather than etiology. Notably, our PLS-DA analysis revealed that metabolic waste profiles align with injury severity rather than etiology (Supplementary Fig. 7 and 8).

Despite the small sample size, our findings highlight the efficacy of cisternal CSF drainage for the clearance of metabolic waste—and specifically of blood-derived substances—in patients with SBI of varying etiology. Our results highlight the clinical relevance of cisternostomy for the removal of metabolic waste after brain injury. However, the narrow range of GCS score made it challenging to establish robust correlations between metabolite levels and recovery metrics, leading to inconsistencies between GCS score and sampling-time analyses. Further studies should also include more comprehensive and continuous metrics to evaluate the progression of recovery for brain injury patients. Nonetheless, our data strongly suggest that cisternostomy aids in the clearance of metabolic waste and supports recovery.

This study utilized non-targeted metabolomics and did not investigate the permeability of the blood-CSF barrier during recovery. Specifically, we lacked data on blood-derived metabolites in healthy subjects or during the very early stages of injury, and thus further investigation warranted. Future research should explore the potential deleterious actions of blood-derived metabolites in the central nervous system, and functional studies could utilize animal models to determine the origins, neuroprotective properties, and potential mechanisms of action of metabolites that increase during recovery.

We hope that future studies can implement similar surgical and experimental designs in large animal models. By including animals without cisternostomy as controls, we will be able to more rigorously test the above three points, thereby providing stronger evidence that the rapid clearance of numerous metabolites is mainly attributable to cisternostomy rather than natural metabolic recovery after brain injury.

## Supplementary Materials

The Supplementary data can be found online at: www.aginganddisease.org/EN/10.14336/AD.2025.0738.

## Data Availability

The datasets generated and/or analyzed in the current study are available from the corresponding authors upon reasonable request.
