# Supplementary material for: Cisternostomy Facilitates Clearance of Metabolic Waste from Cerebrospinal Fluid in Patients with Severe Brain Injury
Source: Aging Dis. 2026 Oct 15;17(6):3427–41. doi: 10.14336/AD.2025.0738 (PMC13653702; doi:10.14336/AD.2025.0738)
Supplement: Supplementary file 1 [file AD-17-6-3427-s.pdf]

SUPPLEMENTARY DATA

Cisternostomy Facilitates Clearance of Metabolic Waste from Cerebrospinal Fluid in Patients with Severe Brain Injury

Weiwei He, Shuai Gao, Lulu Du, Tangrui Han, Di Yao, Hao Wu, Qiang Li, Feixiang Li, Woo-ping Ge, Yonghong Wang

© 2025. He W et al. Published online at http://www.aginganddisease.org/EN/10.14336/AD.2025.0738

SUPPLEMENTARY DATA

Supplementary Table 1

Patient ID	Age	Sex	Etiology	GCS Pre-op	GCS Post-op
1	69	Female	SAH	3	15
2	30	Male	HICH	7	11
3	49	Male	CT	3	11
4	48	Male	CT	3	8
5	38	Male	HICH	3	7
6	65	Female	SAH	6	6
7	34	Male	HICH	3	10
8	34	Female	SAH	3	11
9	56	Female	SAH	8	10
10	34	Male	HICH	10	13
11	58	Female	CT	6	15
12	60	Male	SAH	12	15
13	69	Female	HICH	4	7
14	47	Male	CT	7	14
15	59	Male	CT	3	3

CT, Craniocerebral trauma. HICH, Hypertensive cerebral hemorrhage.
SAH, Aneurysmal subarachnoid hemorrhage. Pre-op, the period preceding surgery. Post-op, the period after surgery.

© 2025. He W et al. Published online at http://www.aginganddisease.org/EN/10.14336/AD.2025.0738

SUPPLEMENTARY DATA

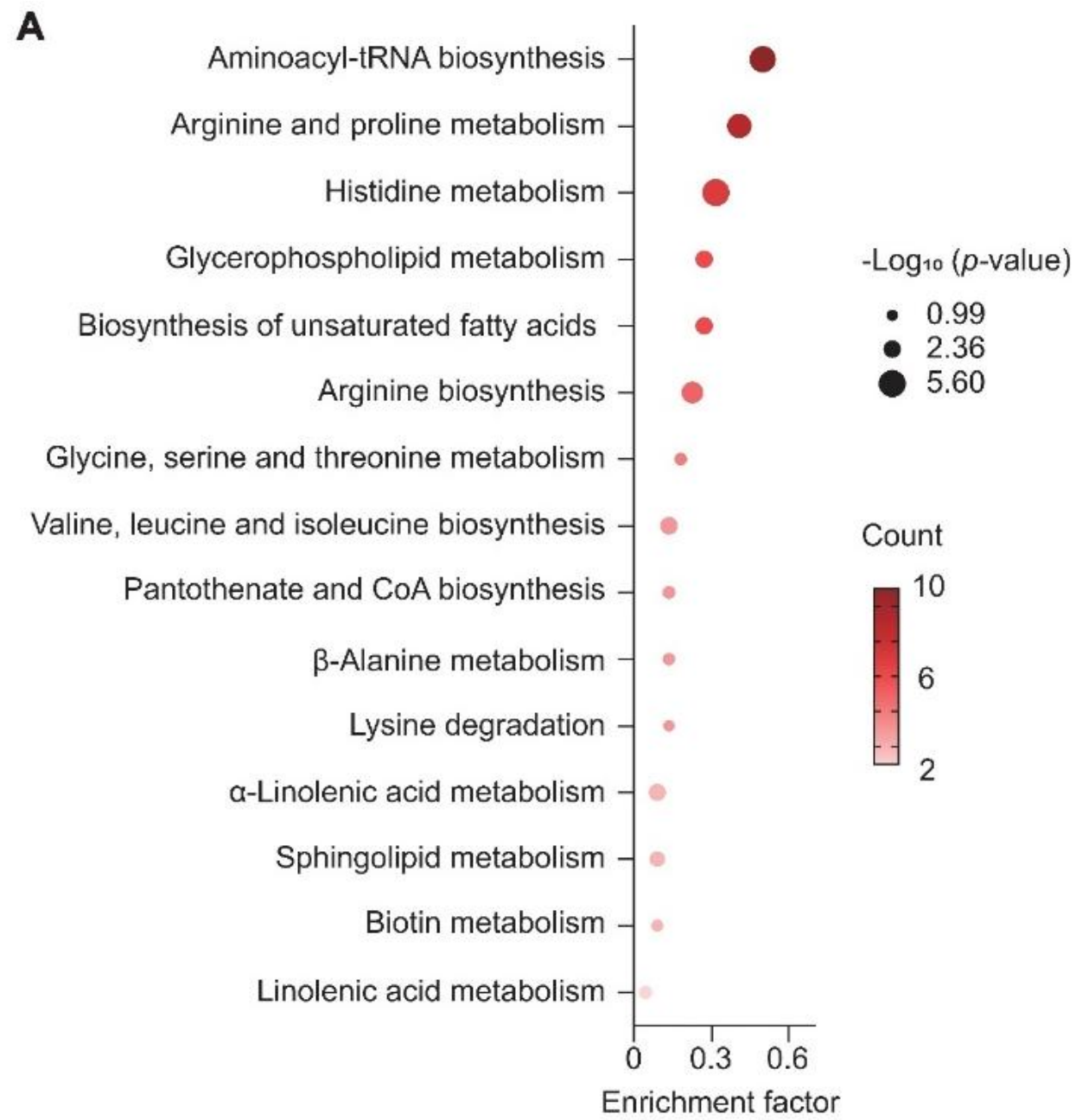

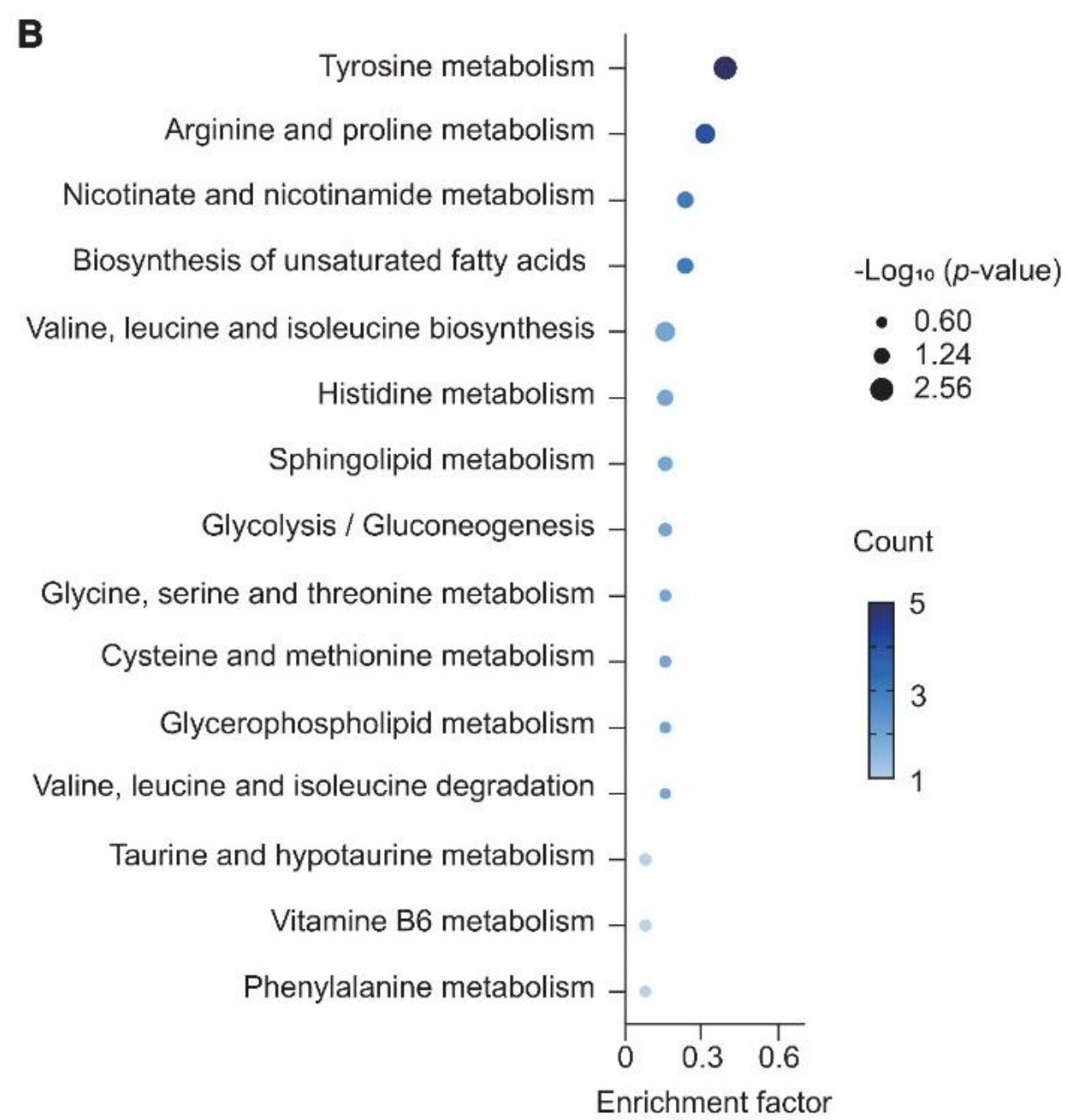

Supplementary Figure 1. Pathway enrichment analysis of the differential metabolomes between blood and LS-CSF. A, B) KEGG-based qualitative enrichment analysis of metabolites in blood (A) and cisternal CSF (B) to identify the most relevant metabolic pathways. Pathways are ranked by adjusted *p*-values and their pathway impact. Circle size represents pathway impact, whereas color indicates the enrichment significance (*p*-value).

© 2025. He W et al. Published online at http://www.aginganddisease.org/EN/10.14336/AD.2025.0738

SUPPLEMENTARY DATA

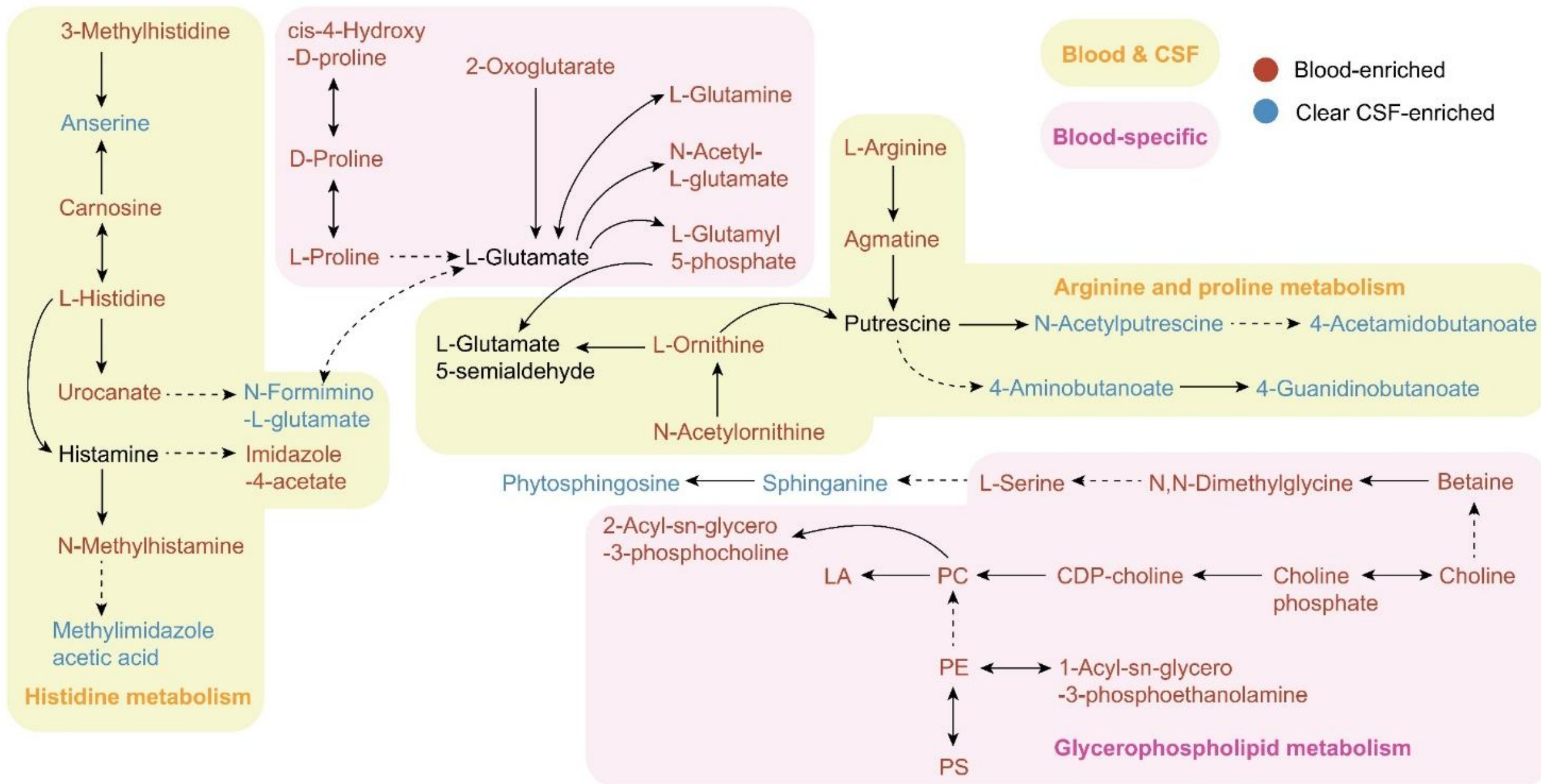

Supplementary Figure 2. Schematic diagram of pathways enriched in blood and CSF. Schematic diagram illustrating the pathways composed of metabolites enriched exclusively in blood, exclusively in LS-CSF, or shared between both blood and LS-CSF. Abbreviations: LA: linoleic acid. PC: phosphatidylcholine. PE: phosphatidylethanolamine. PS: phosphatidylserine. CDP-choline: Cytidine-5'-diphosphate choline.

© 2025. He W et al. Published online at http://www.aginganddisease.org/EN/10.14336/AD.2025.0738

SUPPLEMENTARY DATA

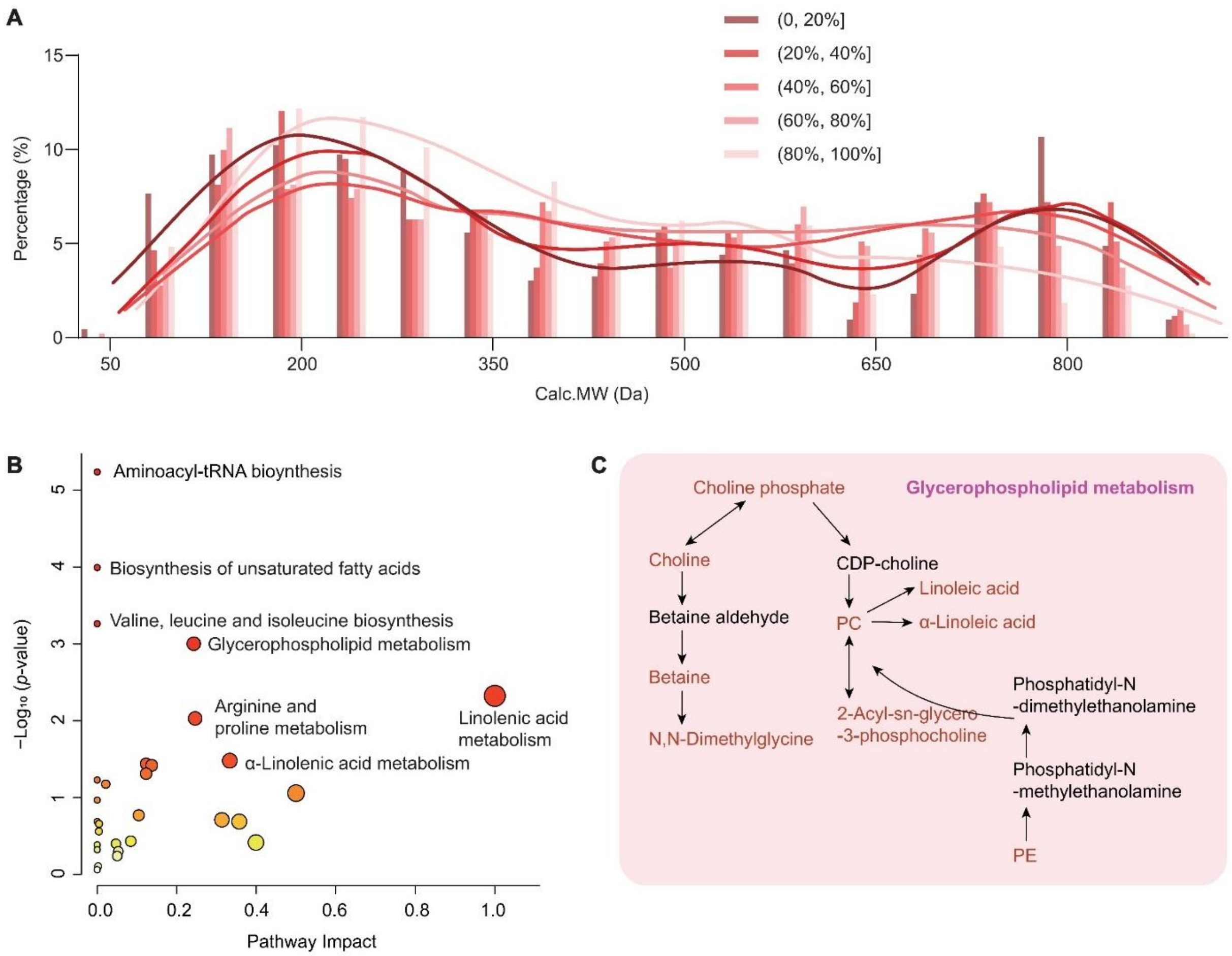

Supplementary Figure 3. Biochemical pathway–based analysis of metabolites enriched in blood. A) Percentile distribution of blood-enriched metabolites based on molecular mass. The percentiles were calculated by determining the number of metabolites within each 50 m/z segment. **B**) KEGG-based qualitative enrichment analysis of pathways using the top 20% of intensity-based blood-enriched metabolites. Pathways were ranked by adjusted p-values and their impact. Circle size represents pathway impact, whereas color indicates enrichment significance (red indicates higher significance). **C**) Schematic pathway diagrams depicting pathways composed of the top 20% of metabolites enriched in blood. Abbreviations in (C): PC, phosphatidylcholine. PE, phosphatidylethanolamine.

© 2025. He W et al. Published online at http://www.aginganddisease.org/EN/10.14336/AD.2025.0738

SUPPLEMENTARY DATA

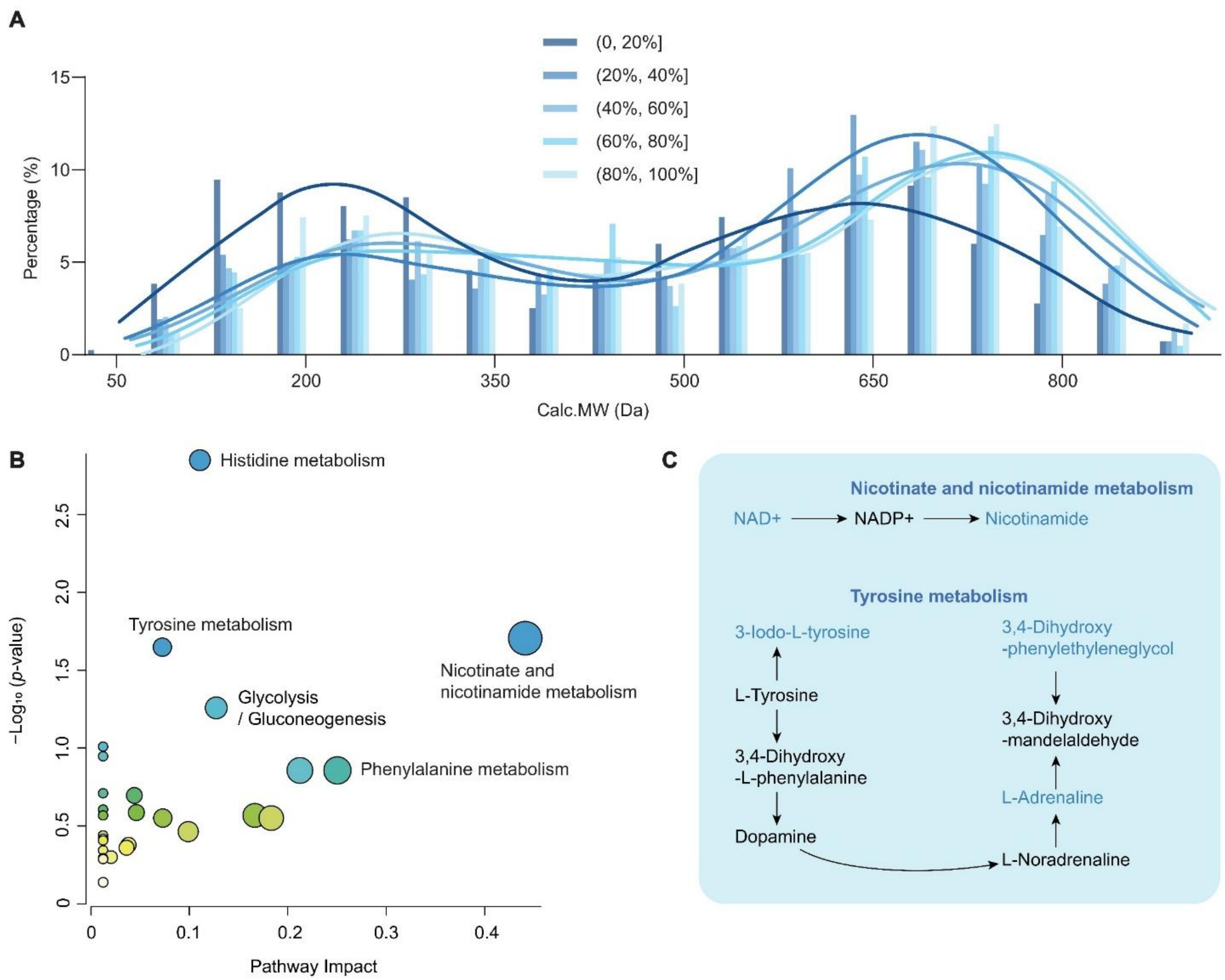

Supplementary Figure 4. Biochemical pathway–based analysis of metabolites enriched in cisternal CSF. A) Percentile distribution of CSF-enriched metabolites based on molecular mass. The percentiles were calculated by determining the number of metabolites within each 50 m/z segment. **B**) KEGG-based qualitative enrichment analysis of pathways using the top 20% of intensity-based CSF-enriched metabolites. Pathways were ranked by adjusted p-values and their impact. Circle size represents pathway impact, whereas color indicates enrichment significance (red indicates higher significance). **C**) Schematic pathway diagrams depicting pathways composed of the top 20% of metabolites enriched in CSF. Abbreviations in (C): NAD, nicotinamide adenine dinucleotide. NADP, nicotinamide adenine dinucleotide phosphate.

© 2025. He W et al. Published online at http://www.aginganddisease.org/EN/10.14336/AD.2025.0738

SUPPLEMENTARY DATA

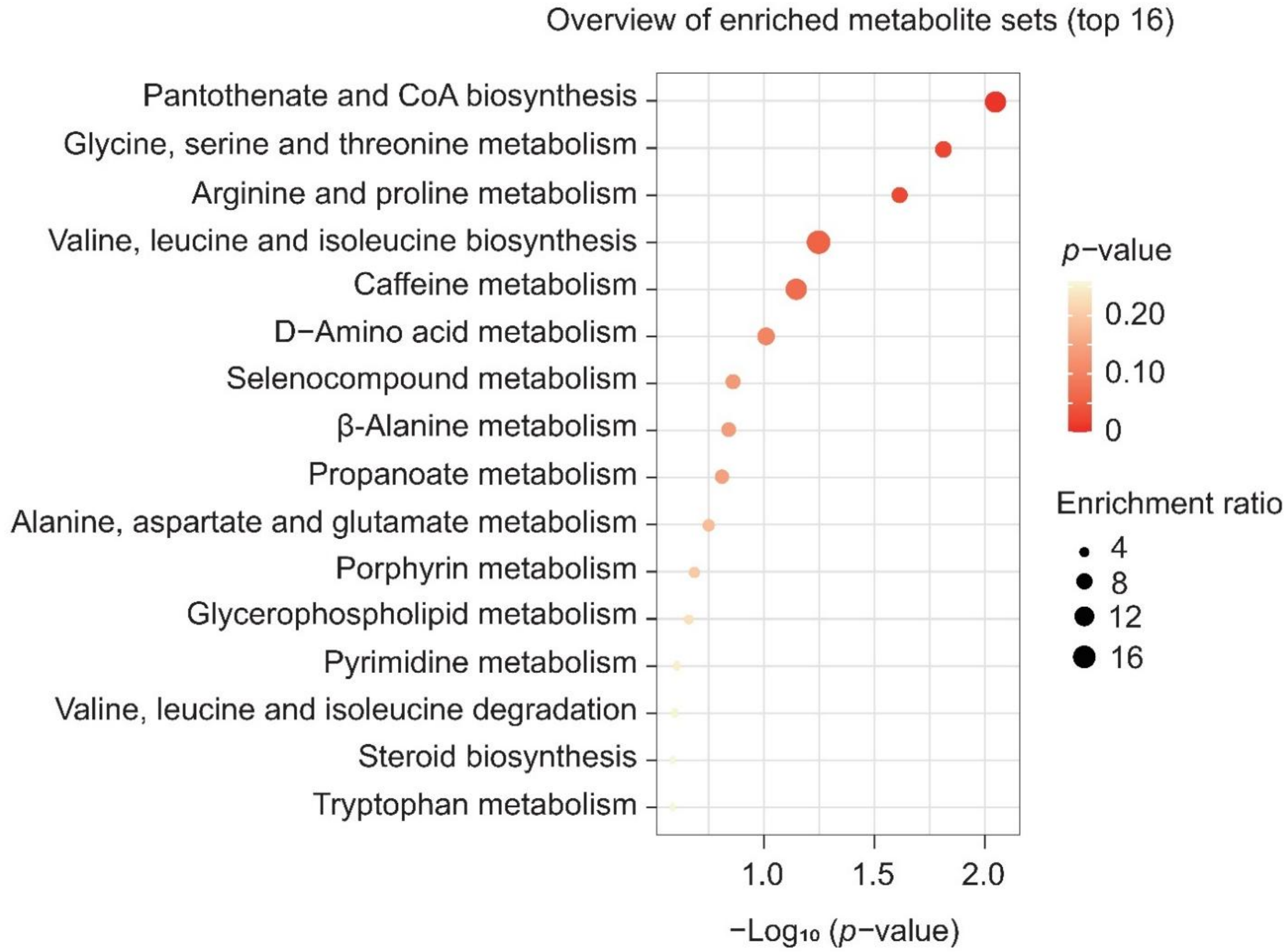

Supplementary Figure 5. Biochemical pathway–based enrichment analysis of the 918 overlapping features between the ES-CSF and blood samples. The bubble plot shows the top 16 metabolic pathways analyzed from the 918 metabolites.

© 2025. He W et al. Published online at http://www.aginganddisease.org/EN/10.14336/AD.2025.0738

SUPPLEMENTARY DATA

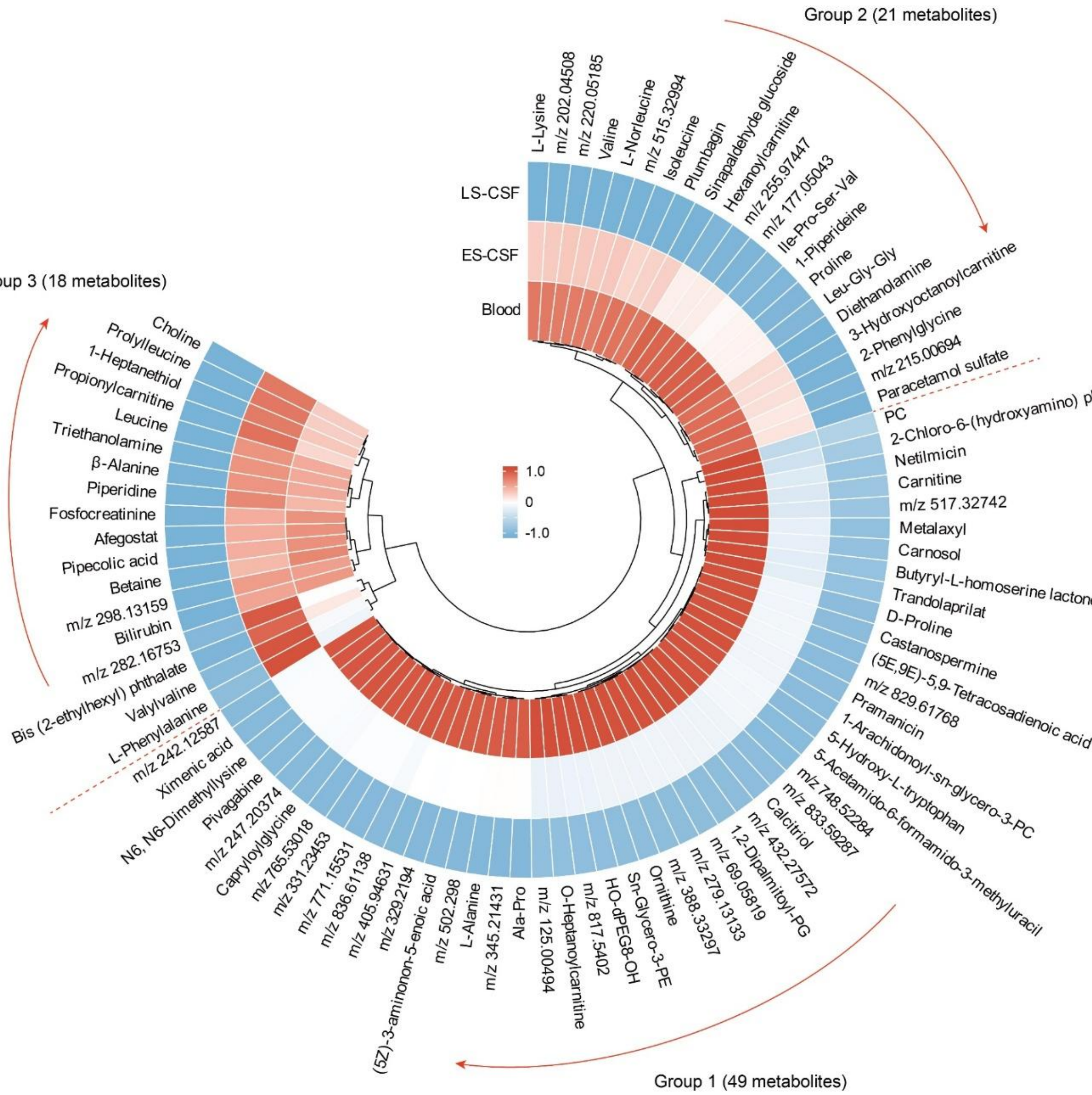

Supplementary Figure 6. Circular heatmap illustrating the distribution of 88 representative metabolites across blood, ES-CSF, and LS-CSF. Metabolites were grouped into three clusters: Group 1 (49 metabolites, from PC to *m/z* 242.12587, clockwise), Group 2 (21 metabolites, from L-lysine to paracetamol sulfate), and Group 3 (18 metabolites, from L-phenylalanine to choline). Group 1 metabolites were predominantly enriched in blood and partially in ES-CSF, but were consistently low in LS-CSF. The central color scale represents standardized metabolite abundance.

© 2025. He W et al. Published online at http://www.aginganddisease.org/EN/10.14336/AD.2025.0738

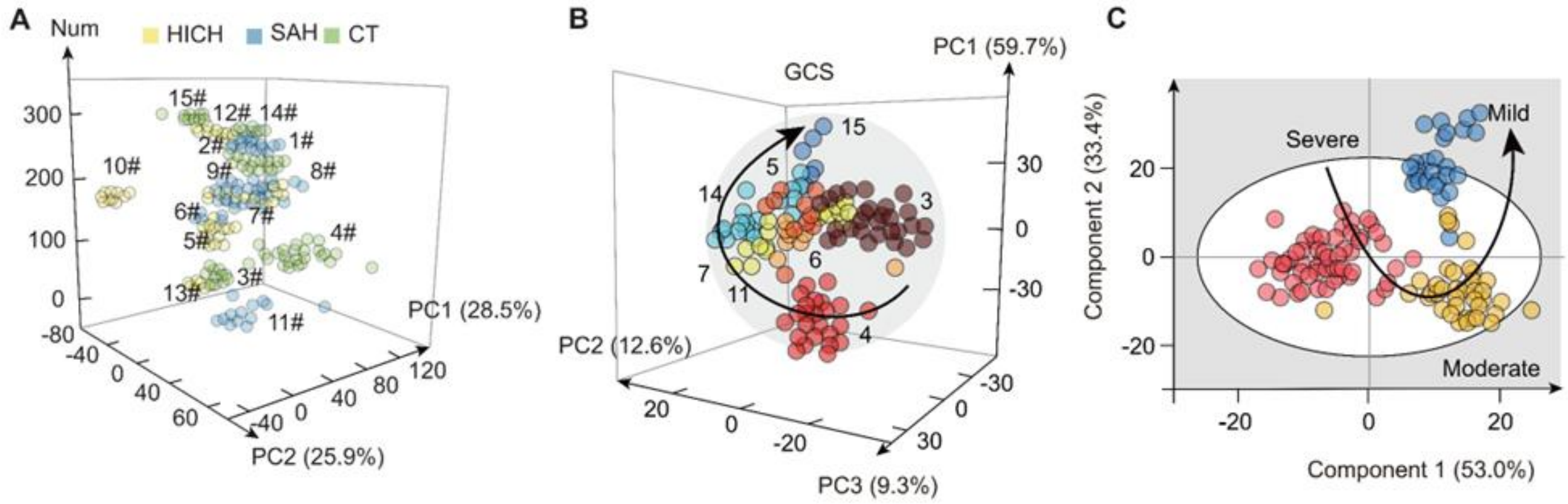

Supplementary Figure 7. Principal component analysis (PCA) of cerebrospinal fluid (CSF) metabolomes classified by patient, Glasgow Coma Scale (GCS) score, and disease severity. A) PCA of the CSF metabolomes of all patients stratified by the etiology of brain injury; CT, Craniocerebral trauma. HICH, Hypertensive cerebral hemorrhage. SAH, Aneurysmal subarachnoid hemorrhage. **B**) PCA of the CSF metabolomes of all patients without etiological stratification, using the GCS score as the grouping criterion; **C**) Partial least squares discriminant analysis (PLS-DA) of the CSF metabolomes of all patients, categorized into severe (GCS score 3–8), moderate (GCS score 9–12), and mild (GCS score 13–15) brain injury groups.

© 2025. He W et al. Published online at http://www.aginganddisease.org/EN/10.14336/AD.2025.0738

SUPPLEMENTARY DATA

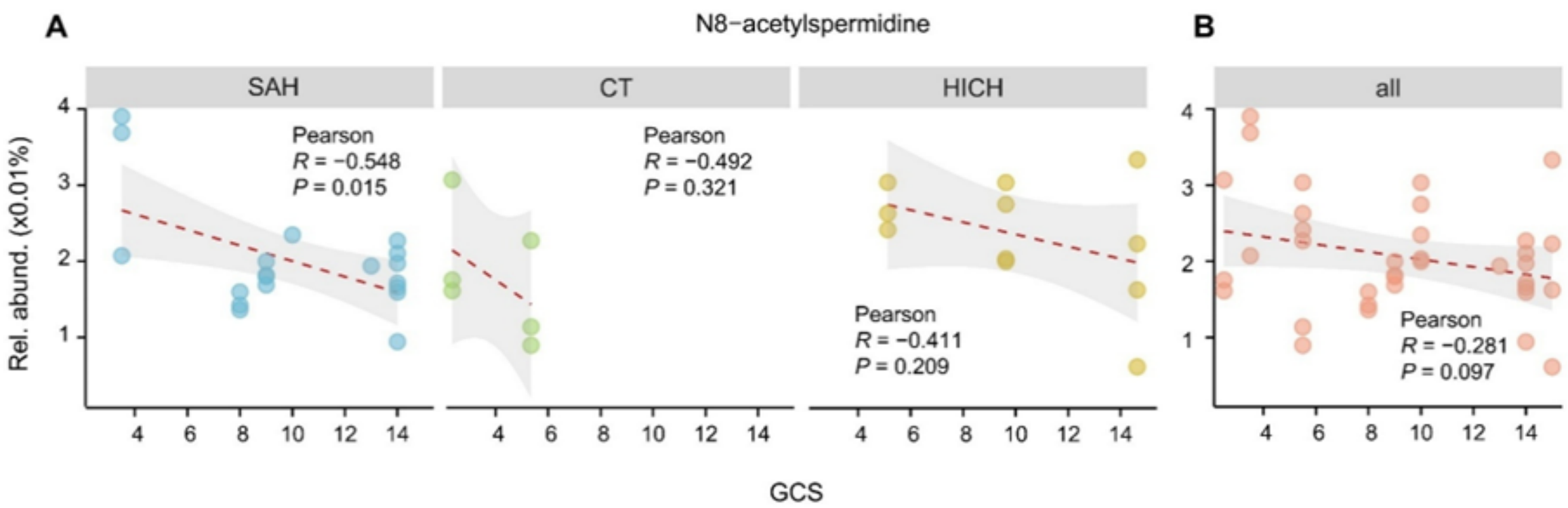

Supplementary Figure 8. N8-acetylspermidine clearance negatively correlates with improvements in Glasgow Coma Scale (GCS) scores across all etiological subgroups. A) Correlation plots between GCS scores and the relative abundance of N8-acetylspermidine were generated for aneurysmal subarachnoid hemorrhage (SAH), cerebral hemorrhage (CT), and hypertensive intracerebral hemorrhage (HICH), displayed from left to right. **B)** A combined analysis of all samples also revealed a negative correlation. The strongest association was observed in SAH ($r = -0.548$, $p = 0.015$). Directionally consistent trends were seen in CT ($r = -0.492$) and HICH ($r = -0.411$), although these did not reach statistical significance, likely due to the small sample size ($n = 5$). Effect sizes (Cohen's f^2) ranged from medium to large (0.17–0.30), supporting biological relevance.

© 2025. He W et al. Published online at http://www.aginganddisease.org/EN/10.14336/AD.2025.0738
